# Prevalence and Associated Factors of Self‐Reported Myopia Among Undergraduate Students in the Northern Region of Bangladesh: A Cross‐Sectional Study

**DOI:** 10.1002/hsr2.72472

**Published:** 2026-05-04

**Authors:** Tajin Ahmed Jisa, Jyoti Shree Roy, Isteaq Kabir Sifat, Md. Sabuj Ali, Farhana Hasan, Md. Golam Hossain, Md. Kaderi Kibria

**Affiliations:** ^1^ Department of Statistics Hajee Mohammad Danesh Science and Technology University Dinajpur Bangladesh; ^2^ Department of Statistics University of Rajshahi Rajshahi Bangladesh

**Keywords:** Bangladesh, cross‐sectional study, Dinajpur City, prevalence, risk factors, self‐reported myopia, undergraduate students

## Abstract

**Background and Aims:**

Myopia is an increasingly important public health concern worldwide, particularly among young adults. While previous research has applied machine learning techniques to identify myopia‐related risk factors, evidence based on traditional statistical models using the same datasets remains limited. This study aimed to estimate the prevalence of self‐reported myopia and examine its associated factors among undergraduate students in the northern region of Bangladesh.

**Methods:**

This secondary analysis utilized cross‐sectional data originally collected between May 17 and June 17, 2024, from 514 undergraduate students enrolled in various academic disciplines. The original study employed a two‐stage sampling technique and collected information using a structured questionnaire covering sociodemographic characteristics, health‐related factors, and lifestyle behaviors. Descriptive statistics were used to estimate prevalence, and binary logistic regression analysis was performed to identify factors associated with myopia. Statistical significance was set at *p* < 0.05.

**Results:**

The prevalence of self‐reported myopia among the participants was 43.2%. Factors significantly associated with myopia included a family history of myopia (OR: 5.12; 95% CI: 3.22–8.13), premature birth (OR: 2.52; 95% CI: 1.25–6.03), visual stress (OR: 3.65; 95% CI: 2.37–5.12), uncertain response to visual stress (OR: 4.15; 95% CI: 2.25–6.76) and steroid use (OR: 2.11; 95% CI: 1.13–4.33). The logistic regression model demonstrated good discriminative ability, with an area under the receiver operating characteristic curve (AUC) of 0.836.

**Conclusion:**

This secondary data analysis indicates a high prevalence of myopia among undergraduate students in the northern region of Bangladesh. Several modifiable and non‐modifiable factors, particularly family history, visual stress, and steroid use, were significantly associated with myopia. These findings underscore the importance of targeted public health strategies and support the use of traditional regression models as a transparent and interpretable alternative to machine learning approaches in epidemiological research.

**Abbreviations**
AUC: area under the curve
CI: confidence interval
DGC: Dinajpur Government College
HSTU: Hajee Mohammad Danesh Science and Technology University
MARMC: M Abdur Rahim Medical College
MICE: multiple imputation by chained equations.
OR: odds ratio
ROC: receiver operating characteristic
VIF: variance inflation factor

## 1 Introduction

Myopia, often known as near‐sightedness, is a visual condition where distant objects appear blurred, but close objects can be seen clearly. It is both a contributing risk factor for vision loss and the primary cause of distance vision impairment globally [1]. The main symptom is difficulty seeing distant objects clearly [2]. The global prevalence of myopia has been increasing at an alarming rate, particularly among young adults and children [3, 4]. Recent global estimates further highlight this trend, with projections suggesting a substantial rise in myopia prevalence worldwide [4, 5]. By 2050, nearly half of the global population is expected to be myopic, which equates to approximately 5 billion people [4]. In Asia, the rates are exceptionally high [6, 7, 8], making myopia a significant public health concern due to its potential to progress to high myopia and associated complications, such as retinal detachment, macular degeneration, and glaucoma [9, 10].

Bangladesh is experiencing a similar upward trend in myopia prevalence, particularly among younger populations. A study conducted among students in the Tangail district reported that nearly 53% were myopic, with approximately 55% of cases occurring among individuals aged 18–24 years [11]. In contrast, the prevalence of myopia in Bangladesh was reported to be only 22.1% in 2004, indicating a substantial increase over time [12]. More recent research among Bangladeshi elementary, middle, and high school students identified associations between myopia and near‐work behaviors, including inappropriate reading and writing posture, with recommended viewing distances greater than 33 cm [13]. Comparable findings have been reported across diverse populations worldwide [6, 14, 15, 16, 17, 18]. Evidence suggests that frequent near‐vision tasks, higher academic demands, limited outdoor activity, and a parental history of myopia are important risk factors [19, 20]. Lifestyle and environmental changes such as prolonged screen exposure, increased engagement in close‐vision activities, and reduced time outdoors have been strongly linked to the rising prevalence of myopia [21, 22, 23]. Although myopia cannot be completely prevented, its progression can be effectively managed through evidence‐based interventions, including orthokeratology lenses [24], atropine eye drops [25], increased outdoor activity [26], multifocal contact lenses [27], and spectacle lenses with peripheral defocus design [28]. A clear understanding of the associated risk factors is therefore essential for designing effective preventive strategies and public health interventions.

In Bangladesh, rapid urbanization and lifestyle transitions have likely contributed to the growing burden of myopia among young adults. Despite this, empirical evidence on myopia prevalence and its associated factors particularly among university students remains limited. Notably, a recent study analyzed self‐reported myopia data among undergraduate students using machine learning techniques to identify risk factors [29]. While machine learning techniques are valuable for pattern recognition and prediction [30], their results may be less transparent and harder to interpret for epidemiological inference, especially when applied to self‐reported outcomes. In contrast, traditional statistical models such as logistic regression offer greater interpretability and are well‐suited for estimating associations and identifying key contributing factors. Given these considerations, the present study aims to estimate the prevalence of self‐reported myopia and examine its associated demographic, health‐related, and lifestyle factors among undergraduate students in Dinajpur City, located in northern Bangladesh. Self‐reported myopia in this study refers to participants' own report of having myopia, with or without a prior clinical diagnosis, including the use of corrective lenses such as glasses or contact lenses. Young adulthood represents a critical period during which myopia may progress, and behaviors such as intensive academic near work and increased screen exposure may substantially influence visual health. By re‐analyzing existing data using a transparent and interpretable statistical framework, this study seeks to generate evidence that can inform public health planning, student health interventions, and behavioral modifications to mitigate the growing burden of myopia in Bangladesh.

## 2 Materials and Methods

### 2.1 Study Design, Population, and Data Source

This study is a secondary analysis of data from a previously conducted cross‐sectional study among undergraduate students in Dinajpur City, located in northern Bangladesh [29]. The original study was carried out between May 17 and June 17, 2024, and employed a two‐stage sampling technique to recruit participants from three academic institutions: Hajee Mohammad Danesh Science and Technology University (HSTU), Dinajpur Government College (DGC), and M Abdur Rahim Medical College (MARMC). These institutions were selected to capture diversity in academic disciplines, including science and technology, medical sciences, and general education, providing a broad perspective on myopia prevalence among undergraduate students. A stratified sampling method was used. Students from each institution formed separate strata, and participants within each stratum were randomly selected using simple random sampling (SRS). The number of participants selected from each stratum was proportionate to its representation in the total population of undergraduate students. Only full‐time undergraduate students who willingly participated were included, and students who declined or were not enrolled full‐time were excluded. The total sample size of 514 was determined using Cochran's single proportion formula [29, 31]. This study was reported in accordance with the Strengthening the Reporting of Observational Studies in Epidemiology (STROBE) guidelines for cross‐sectional studies.

### 2.2 Outcome Variable

The outcome variable of interest was self‐reported myopia status. Participants were asked whether they had been diagnosed with myopia, and responses were categorized as “Yes” (coded as 1) or “No” (coded as 0). The diagnosis was based on participants' self‐report, which may reflect either a prior clinical diagnosis by an eye care professional or personal awareness of myopia, including the use of corrective lenses such as glasses or contact lenses. No objective clinical verification was performed in this study.

### 2.3 Demographic and Health Variables

The demographic variables included age, gender, residence (urban or rural), living conditions (hall, mess, home, other), and economic status (poor, middle‐income, rich). Health‐related variables included family history of myopia, premature birth status, eye pain, headaches, visual stress, redness of the eye, severe eye injury, other eye disorders, and specific conditions like glaucoma and retinal detachment. Participants were asked whether they were born prematurely and whether they had experienced eye pain, with responses categorized as “yes” or “no.” They also reported the frequency and severity of headaches, particularly those related to eye strain or prolonged screen time, and symptoms of visual stress, such as blurred vision, eye strain, or discomfort during visual tasks, as well as instances of eye redness; these variables were categorized as “yes,” “no,” and “sometimes”. Participants were asked about any history of severe eye injuries. Additionally, glaucoma was considered present if clinically diagnosed by an ophthalmologist, and participants reported whether they had been diagnosed with retinal detachment, with both conditions categorized as “yes” or “no”.

### 2.4 Lifestyle Factors

Participants were asked about their dietary intake, sleep duration, screen light exposure, use of steroid medications, time spent on outdoor activities, usage of electronic devices, engage in low light work, and time spent on reading, writing, or homework. Dietary intake was categorized into three broad groups to help participants self‐assess their overall eating habits: “poor” for a diet with low nutritional value, “middle” for a moderately balanced diet, and “rich” for a diet high in nutritional value. Participants selected the category that best described their typical diet. Screen light exposure was evaluated based on participants' familiarity with the term, categorized as “yes” if they were aware of it and “no” if they were not. Screen‐related behaviors were assessed using proxy measures, including self‐reported duration of electronic device use and awareness of screen light exposure. The latter reflects participants' awareness rather than objective exposure. Time spent on outdoor activities was defined as participation in outdoor activities such as sports or walking, categorized as “yes” for more than 1 h per day and “no” for less than 1 h. Spending too much time on electronic devices was assessed based on the duration of use for non‐academic purposes, categorized as “yes” for usage exceeding 4 h per day and “no” for less than 2 h per day. Additionally, participants' involvement in daily sports activities and their mobile phone usage were recorded. For variables such as visual stress and screen exposure, responses included “Yes,” “No,” and “Maybe.” The “Maybe” category represents participants' uncertainty or partial experience of the condition. It was retained to capture uncertainty or partial exposure and was analyzed as a separate category to avoid misclassification bias. Combining it with “Yes” or “No” could obscure potentially meaningful differences in associations.

### 2.5 Data Processing

Missing values in the dataset were handled using Multiple Imputation by Chained Equations (MICE) as in the original dataset (Table S1). Multicollinearity among predictor variables was assessed using the variance inflation factor (VIF), with all VIF values < 5. Rank correlations were visualized using a correlation matrix to ensure independence of variables (Figure S1).

### 2.6 Statistical Analysis

Descriptive statistics were used to summarize participants' demographic, health, and lifestyle characteristics. Categorical variables were presented as frequencies and percentages. Associations between explanatory variables and myopia status were assessed using the chi‐square test. Variables showing significant associations in bivariate analyses were included in the multivariable binary logistic regression model to identify independent factors associated with myopia. In addition to statistical significance in bivariate analyses, variables were also considered based on their epidemiological relevance as reported in previous literature. Although a parsimonious model was prioritized to avoid overfitting, key variables were carefully evaluated as potential confounders. Although this approach may omit variables that are not statistically significant in bivariate analysis, it allows a parsimonious model and avoids overfitting, given the sample size. Multicollinearity among independent variables was assessed using VIFs, and no significant multicollinearity was observed (VIF < 5). The strength of associations was expressed as odds ratios (ORs) with 95% confidence intervals (CIs). The discriminative ability of the final logistic regression model was evaluated using a receiver operating characteristic (ROC) curve. All statistical tests were two‐sided, and a *p*‐value < 0.05 was considered statistically significant. Analyses were conducted using IBM SPSS Statistics (version 26.0) and R software (version 4.2.2), following the SAMPL guidelines for statistical reporting.

### 2.7 Ethics Approval and Consent to Participate

This study is approved by the Institutional Animal, Medical Ethics, Biosafety, and Biosecurity Committee (IAMEBBC), University of Rajshahi, Rajshahi‐6205, Bangladesh, with approval number: 215/320/(69)|AMEBBC/|BSc. Written informed consent was obtained from all participants before their involvement in the study. Each participant was provided with a comprehensive explanation of the research objectives, procedures, potential risks, and benefits to ensure they fully understood the nature of their participation.

## 3 Results

### 3.1 Demographic and Health Profiles of the Participants

The demographic characteristics of the study participants are detailed in Table 1. The majority of participants are aged between 21 and 23 years (55.1%), with a substantial portion aged 24 years or older (40.7%). The gender distribution indicates a higher proportion of females (62.6%) compared to males (37.4%). A majority of participants reside in urban areas (55.6%) as opposed to rural areas (44.4%). Concerning residential status, a significant number live in halls (43.6%), followed by those in messes (31.5%) and homes (22.4%), with a small fraction in other accommodations (2.3%). The economic status reveals that most participants come from middle‐income families (87.7%), with fewer from poor (9.1%) or rich (3.1%) families. More than half of the participants (55.1%) have a family history of myopia, which could be important for investigating genetic or environmental factors related to the condition. Only a small percentage of participants (7.4%) were born prematurely, while the majority (92.6%) were not. These findings provide a thorough overview of the participants' demographics, living conditions, and pertinent health background.

**Table 1:** Demographic information of the study participants (*n* = 514).

Variable		*n*	Percentage (%)
Age	< = 20 years	22	4.3
	21–23 years	283	55.1
	> = 24 years	209	40.7
Gender	Male	192	37.4
	Female	322	62.6
Living place	Rural	228	44.4
	Urban	286	55.6
Current residual status	Hall	224	43.6
	Mess	162	31.5
	Home	115	22.4
	Others	12	2.3
Family income	Poor	47	9.1
	Middle	451	87.7
	Rich	16	3.1
Have any family members who also suffer from myopia	Yes	283	55.1
	No	231	44.9
Were you born prematurely	Yes	38	7.4
	No	476	92.6

As shown in Table 2, myopia is prevalent among undergraduate students, with notable associations between myopia and various health conditions. A high proportion of students with eye pain (88.5%), visual stress (88.0%), and other eye disorders (88.0%) suffer from myopia, suggesting these factors may contribute to its prevalence. Conversely, the prevalence of myopia is slightly lower among participants with diabetes (80.0%), smoking addiction (80.7%), and severe eye injuries (77.3%) compared to those without these conditions. Additionally, those with frequent headaches (84.8%) and redness in the eyes (86.5%) show a higher prevalence of myopia, while those with retinal detachment have a significantly lower prevalence (60.0%) compared to participants without this condition (83.1%). Overall, these findings suggest that myopia is more common among students experiencing eye‐related symptoms and conditions, highlighting the need for targeted eye health interventions.

**Table 2:** Prevalence and risk factors of myopia among the undergraduate students.

Variables		Myopia status		Total
		Yes (*n* = 222) *n* (%)	No (*n* = 292) *n* (%)	
Suffer from eye pain	Yes	177 (88.5)	23 (11.5)	200
	No	247 (78.6)	67 (21.4)	314
Suffer from diabetes	Yes	4 (80.0)	1 (20.0)	5
	No	421 (82.7)	88 (17.3)	509
Addicted smoking	Yes	46 (80.7)	11 (19.3)	57
	No	379 (82.9)	78 (17.1)	457
Suffer from headache	Yes	196 (84.8)	35 (15.2)	231
	No	122 (79.7)	31 (20.3)	153
	Sometimes	107 (82.3)	23 (17.7)	130
Suffer from visual stress	Yes	162 (88.0)	22 (12.0)	184
	No	133 (78.7)	36 (21.3)	169
	Sometimes	130 (80.7)	31 (19.3)	161
Any redness in the eyes	Yes	64 (86.5)	10 (13.5)	74
	No	310 (82.7)	65 (17.3)	375
	Sometimes	51 (78.5)	14 (21.5)	65
Any severe eye injury	Yes	17 (77.3)	5 (22.7)	22
	No	385 (83.3)	77 (16.7)	462
	Maybe	23 (76.7)	7 (23.3)	30
Any other eye disorder	Yes	44 (88.0)	6 (12.0)	50
	No	381 (82.1)	83 (17.9)	464
Having glaucoma	Yes	9 (81.8)	2 (18.2)	11
	No	416 (82.7)	87 (17.3)	503
Having retinal detachment	Yes	6 (60.0)	4 (40.0)	10
	No	419 (83.1)	85 (16.9)	504

### 3.2 Lifestyle Factors of the Respondents

The results presented in Table 3 show the distribution of various lifestyle factors among the respondents that contributed to the development of myopia. The prevalence of myopia is higher among those with a moderately balanced diet (83.9%) compared to those with a diet low in nutritional value (78.7%) or high in nutritional value (76.2%), suggesting a possible association between the quality of dietary intake and myopia. Sleep duration also shows a notable association, with a higher prevalence of myopia among those sleeping 6–8 h (85.0%) compared to those sleeping above 8 h (71.4%). Participants exposed to screen light exhibit a significantly higher prevalence of myopia (88.7%) than those not exposed (75.0%). Insufficient time spent on outdoor activities is associated with a higher prevalence of myopia (87.0%), while those who spend excessive time on electronic devices also have a high prevalence (86.3%). Furthermore, the prevalence of myopia is higher among those engaged in low light work (84.2%) and those spending 3–4 h on reading, writing, or homework (87.8%). Reduced participation in daily sports activities correlates with a higher prevalence of myopia, particularly among those with no activity (86.9%). Additionally, participants who use mobile phones for 2–3 h daily (84.0%) or above 4 h (82.5%) show a higher prevalence of myopia compared to those who use mobile phones for less than 1 h (73.1%).

**Table 3:** Daily life activities contributing to myopia development among participants.

Variables		Myopia status		Total
		Yes (*n* = 222) *n* (%)	No (*n* = 292) *n* (%)	
Kind of dietary intake	Poor	70 (78.7)	19 (21.3)	89
	Middle	339 (83.9)	65 (16.1)	404
	Rich	16 (76.2)	5 (23.8)	21
Sleeping time	4–6 h	48 (84.2)	9 (15.8)	57
	6–8 h	317 (85.0)	56 (15.0)	373
	Above 8 h	60 (71.4)	24 (28.6)	84
Screen light exposure	Yes	250 (88.7)	32 (11.3)	282
	No	117 (75.0)	39 (25.0)	156
	Maybe	58 (76.3)	18 (23.7)	76
Certain steroid medication	Yes	47 (81.0)	11 (19.0)	58
	No	378 (82.9)	78 (17.1)	456
Spending insufficient time on outdoor activities	Yes	207 (87.0)	34 (13.0)	238
	No	218 (79.0)	58 (21.0)	276
Spending too much time on electronic devices	Yes	252 (86.3)	40 (13.7)	292
	No	78 (73.6)	28 (26.4)	106
	Maybe	95 (81.9)	21 (18.1)	116
Low‐light work	Yes	160 (84.2)	30 (15.8)	190
	No	265 (81.8)	59 (18.2)	324
Spent in reading, writing, or on homework	Less than 1 h	76 (73.8)	27 (26.2)	103
	1–2 h	109 (81.3)	25 (18.7)	134
	3–4 h	137 (87.8)	19 (12.2)	156
	Greater than 4 h	103 (85.1)	18 (14.9)	121
Daily sports activities	Less than half hour	97 (81.5)	22 (18.5)	119
	0.5–1 h	57 (75.0)	19 (25.0)	76
	1–2 h	37 (75.5)	12 (24.5)	49
	Greater than 3 h	8 (80.0)	2 (20.0)	10
	No activity	226 (86.9)	34 (13.1)	260
Average time spent using a mobile phone	Below 1 h	19 (73.1)	7 (26.9)	26
	1–2 h	48 (82.8)	10 (17.2)	58
	2–3 h	173 (84.0)	33 (16.0)	206
	Above 4 h	174 (82.5)	37 (17.5)	211
	Greater than 3 h	11 (84.6)	2 (15.4)	13

### 3.3 Associated Factors Identification

**Figure 1:**
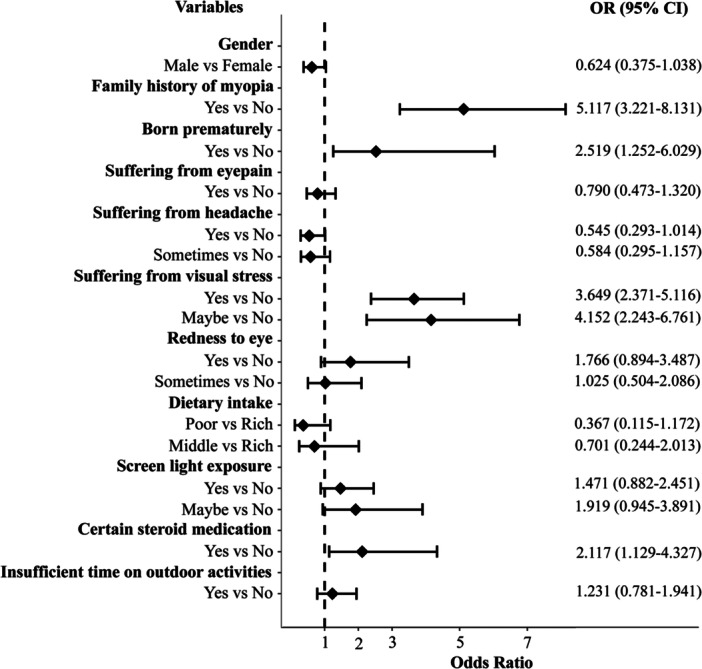
The odds ratio and its 95% confidence interval were presented by this figure, whereas the *x*‐axis indicates the odds ratio and the *y*‐axis presents the associated factors of myopia.

Binary logistic regression was conducted to identify the associated factors for myopia, and the results are presented in Figure 1. Prior to this analysis, an association analysis was performed among the study variables and other factors revealed 12 factors significantly associated with myopia (see Table S2). These factors were subsequently included in the logistic regression analysis. Participants with a family history of myopia were over 5 times more likely to develop myopia compared to those without a family history (OR: 5.117, 95% CI: 3.221–8.131). Being born prematurely also more than doubled the risk of developing myopia (OR: 2.519, 95% CI: 1.252–6.029). Having eye pain, headaches, or redness of the eye did not significantly affect the likelihood of developing myopia. However, participants who experienced visual stress were about 3.6 times more likely to have myopia (OR: 3.649, 95% CI: 2.370–5.116), and those who answered “Maybe” for visual stress were more than 4 times more likely to develop it (OR: 4.152, 95% CI: 2.246–6.760). Screen light exposure wasn't strongly associated with myopia, but those who answered “Maybe” for screen light exposure were almost 2 times more likely to develop it (OR: 1.919, 95% CI: 0.945–3.897). Participants taking certain steroid medications were about 2 times more likely to have myopia (OR: 2.110, 95% CI: 1.129‐4.327). Dietary intake, insufficient time spent on outdoors was not statistically significant.

**Figure 2:**
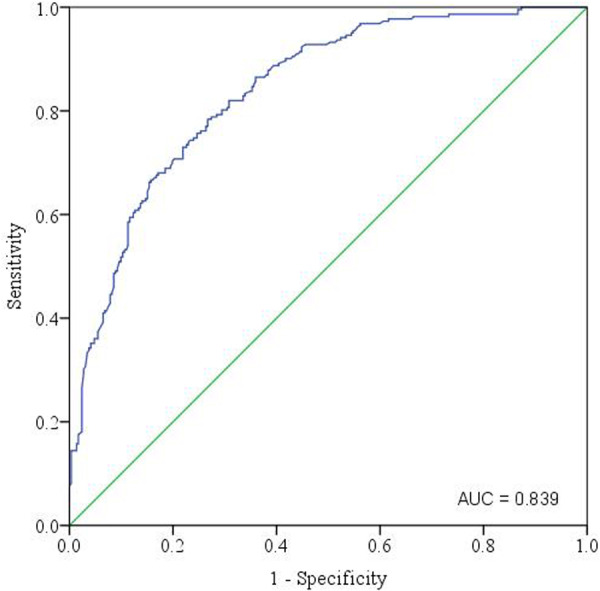
This curve shows the diagnostic performance of a binary logistic regression model for myopia risk factors, with the blue line representing the model and the green diagonal indicating a random classifier.

The discriminatory performance of the fitted binary logistic regression model was determined using the area under the curve (AUC). The ROC curve shown represents the diagnostic performance of this model in assessing risk factors for myopia. The *x*‐axis displays the false positive rate (1 ‐ specificity), while the *y*‐axis shows the true positive rate (sensitivity). The AUC value for this ROC curve is 0.836, indicating that the model has a very good ability to discriminate between individuals with and without myopia (Figure 2).

## 4 Discussion

The present study aimed to determine the prevalence of self‐reported myopia and its associated factors among undergraduate students in Dinajpur City, northern Bangladesh, using a traditional binary logistic regression framework applied to secondary cross‐sectional data. The observed prevalence of myopia (43.2%) indicates a substantial burden among this population and is consistent with the increasing trends reported in Bangladesh and other South Asian settings [13, 32]. Although many previous studies have focused on children and adolescents, these findings remain relevant as myopia often develops earlier in life and shares common behavioral and environmental risk factors across age groups, including university students. This high prevalence likely reflects the combined effects of intensive academic demands, prolonged near‐work activities, and lifestyle transitions characteristic of university students.

A strong and consistent association was observed between family history of myopia and myopia status, highlighting the role of genetic susceptibility. This finding is supported by extensive evidence demonstrating hereditary influences on myopia development across diverse populations [4, 33, 34, 35]. Importantly, family history was also identified as a key predictor in the prior machine learning–based analysis of the same dataset [29], suggesting that this factor is robust to analytical approach and remains a critical determinant of myopia risk regardless of modeling strategy. Gender and residential background were also significantly associated with myopia in the present analysis, with females and urban students exhibiting a higher likelihood of myopia. These findings are consistent with earlier regional and international studies [35, 36] and may reflect differences in study‐related near work, screen exposure, and outdoor activity. While the machine‐learning study identified excessive screen time and insufficient outdoor activities as important predictors [29], our regression‐based findings complement these results by quantifying their associations and highlighting the role of visual stress and environmental exposure in a more interpretable epidemiological framework. Specifically, females and urban students showed a higher susceptibility to myopia, underscoring the influence of environmental and lifestyle factors. These findings align with research that associates urban living and reduced outdoor activities with increased myopia risk [37]. The logistic regression analysis provided valuable insights into the risk factors, including family history of myopia, premature birth, visual stress, screen light exposure, and the use of certain steroid medications. Specifically, males exhibited a lower risk compared to females, aligning with findings from other studies that explore gender differences in myopia susceptibility [38]. Premature birth emerged as another significant risk factor, consistent with literature that links early birth to various developmental challenges, including ocular conditions [39, 40, 41]. Visual stress was another critical factor, significantly associated with higher myopia prevalence, possibly due to prolonged visual strain [42]. A study found the link between psychological stress in childhood and the development of myopia [43]. Interestingly, our study found that poorer dietary intake was associated with a lower likelihood of myopia, which is a counterintuitive finding. This result should be interpreted with caution, as the relationship between nutrition and myopia remains complex and not fully understood [36, 44]. One possible explanation is residual confounding, where dietary patterns may be linked with other lifestyle factors such as socioeconomic status, screen time, or outdoor activity [45]. Additionally, students reporting a richer diet may have more urbanized lifestyles, which are themselves associated with higher myopia prevalence. The lack of a significant association between outdoor activity and myopia in this study contrasts with established evidence [46, 47]. This discrepancy may be due to the use of self‐reported and dichotomized measures, which may not adequately capture duration, intensity, or cumulative exposure to outdoor light. Self‐reported dietary assessment may also introduce misclassification. This counterintuitive finding may also reflect lifestyle clustering, where students with higher‐quality diets may simultaneously engage in more intensive academic activities, prolonged screen exposure, and reduced outdoor time [48]. Such interrelated behaviors may confound the observed relationship between diet and myopia. Existing literature on nutrition and myopia is limited and mixed, with some studies suggesting that high glycemic diets and metabolic factors may influence ocular growth, although evidence remains inconclusive [49, 50]. Although significant screen light exposure was not directly associated with myopia, participants who responded “Maybe” to screen light exposure were nearly twice as likely to develop myopia [51]. The “Maybe” category represents participants' uncertainty or partial experience of the condition. We retained this category separately to preserve this information and avoid misclassification. Combining it with “Yes” or “No” could obscure potentially meaningful differences in associations, and may explain why the “Maybe” category occasionally shows stronger associations than “Yes.” This suggests that uncertain or moderate screen use habits could still influence eye health [52]. However, screen‐related variables in this study were measured using indirect indicators rather than precise estimates of daily screen time or differentiation between academic and non‐academic use. This limitation may affect the accuracy of capturing the true relationship between screen exposure and myopia. Previous studies have found a significant association between prolonged screen time and myopia development, particularly in children and adolescents, indicating the need for further investigation into the impact of screen habits on eye health [51, 53]. The use of certain steroids was found to be associated with higher odds of myopia in this study [54]. However, this finding should be interpreted with caution, as steroid use is not widely established as a primary risk factor for simple myopia in the ophthalmic literature [55]. Previous studies have primarily linked steroid use to ocular conditions such as increased intraocular pressure, cataract formation, and, in some cases, transient refractive changes rather than permanent myopia [56, 57, 58]. The observed association in our study may reflect underlying confounding factors or self‐reported medication use patterns. This association should be interpreted cautiously, as it may reflect underlying health conditions, reporting bias, or unmeasured confounding rather than a direct causal relationship. Therefore, further longitudinal and clinically validated studies are needed to better understand the potential relationship between steroid use and myopia. Conversely, factors like eye pain, headaches, and eye redness were not significantly associated with myopia, suggesting these symptoms do not directly contribute to its development in our study population [59]. The model demonstrated strong discriminatory power (AUC = 0.836), indicating good model performance and supporting the reliability of the identified associations. Compared with machine learning approaches, traditional regression models provide greater transparency and interpretability, particularly when analyzing self‐reported outcomes. While machine learning techniques are advantageous for prediction and handling complex interactions, regression‐based models remain valuable for epidemiological inference and public health decision‐making. Together, findings from both analytical approaches offer complementary insights into the multifactorial etiology of myopia.

## 5 Limitations and Recommendations

This study has several limitations. Its cross‐sectional design limits the ability to infer causality. The focus on undergraduate students in Dinajpur City may restrict the generalizability of the results to other populations. Additionally, myopia status was based on self‐report, which may reflect either a prior clinical diagnosis by an eye care professional or personal awareness, including the use of corrective lenses such as glasses or contact lenses. No objective clinical verification was performed. This introduces the possibility of misclassification bias, as some participants may incorrectly report their myopia status or be unaware of their refractive errors, potentially leading to under‐ or overestimation of prevalence and associations with risk factors. Furthermore, the multivariable logistic regression model included only variables that were significant in bivariate analyses. This approach may have omitted other important potential confounders, such as age, gender, screen time, working hours, and outdoor activity, which could influence the observed associations. Furthermore, the selection of variables for the multivariable model based primarily on bivariate significance may have resulted in residual confounding. Important variables such as age, gender, screen time, and outdoor activity may still influence the observed associations despite not being statistically significant in preliminary analyses. Future studies should consider including theoretically relevant variables regardless of statistical significance to better control for confounding. Despite these limitations, the study provides valuable insights into the prevalence and risk factors of myopia among undergraduate students in Dinajpur City, Bangladesh. Future studies should incorporate objective vision assessments and include a broader range of potential confounders to strengthen the modeling of factors associated with myopia and improve the accuracy of findings.

## 6 Conclusions

This study analysis demonstrates that myopia affects a substantial proportion of undergraduate students in northern Bangladesh, with nearly half of the participants reporting the condition. Key risk factors identified include family history of myopia, premature birth, visual stress, screen light exposure, and use of steroid medications. In contrast, symptoms such as eye pain, headaches, or redness, as well as dietary intake and limited outdoor activity, were not significantly associated with myopia risk. ROC curve analyses indicate that these factors collectively provide a reliable framework for understanding myopia risk in this population. These findings highlight the importance of early identification and targeted interventions, particularly for students at higher risk due to family history or premature birth. Public health initiatives should focus on raising awareness and promoting lifestyle modifications to mitigate myopia progression. Further research is warranted to clarify the roles of diet and screen light exposure in myopia development and to guide evidence‐based preventive strategies.

## Author Contributions

**Tajin Ahmed Jisa:** conceptualization, methodology, data curation, formal analysis, writing – original draft, visualization. **Jyoti Shree Roy:** methodology, software, formal analysis. **Isteaq Kabir Sifat:** software, methodology, writing – original draft. **Md. Sabuj Ali:** investigation, methodology, project administration. **Farhana Hasan:** writing – review and editing; validation. **Md. Golam Hossain:** validation, investigation, writing – review and editing. **Md. Kaderi Kibria:** conceptualization, methodology, software, investigation, validation, supervision, project administration, writing – review and editing.

## Funding

The authors have nothing to report.

## Disclosure

The lead author, Md. Kaderi Kibria, affirms that this manuscript is an honest, accurate, and transparent account of the study being reported; that no important aspects of the study have been omitted; and that any discrepancies from the study as planned (and, if relevant, registered) have been explained.

## Conflicts of Interest

The authors declare no conflicts of interest.

## Supporting information

Supplementary File

## Data Availability

The data that support the findings of this study are available in the supporting material of this article.
