# Supplementary material for: Prevalence and Associated Factors of Self‐Reported Myopia Among Undergraduate Students in the Northern Region of Bangladesh: A Cross‐Sectional Study
Source: Health Sci Rep. 2026 May 4;9(5):e72472. doi: 10.1002/hsr2.72472 (PMC13139634; doi:10.1002/hsr2.72472)
Supplement: Supplementary file 1 — Supplementary File [file HSR2-9-e72472-s001.docx]

Prevalence and Associated Factors of Self-Reported Myopia among Undergraduate Students in the Northern Region of Bangladesh

**Table S1:** Number of missing responses for each variable.

| S/N | Variables | No. of missing values |
| --- | --- | --- |
| 1. | Are you born prematurely? | 3 |
| 2. | Do you suffer from Diabetes? | 7 |
| 3. | Do you have glaucoma ? | 11 |
| 4. | Do you have retinal detachment? | 2 |

| 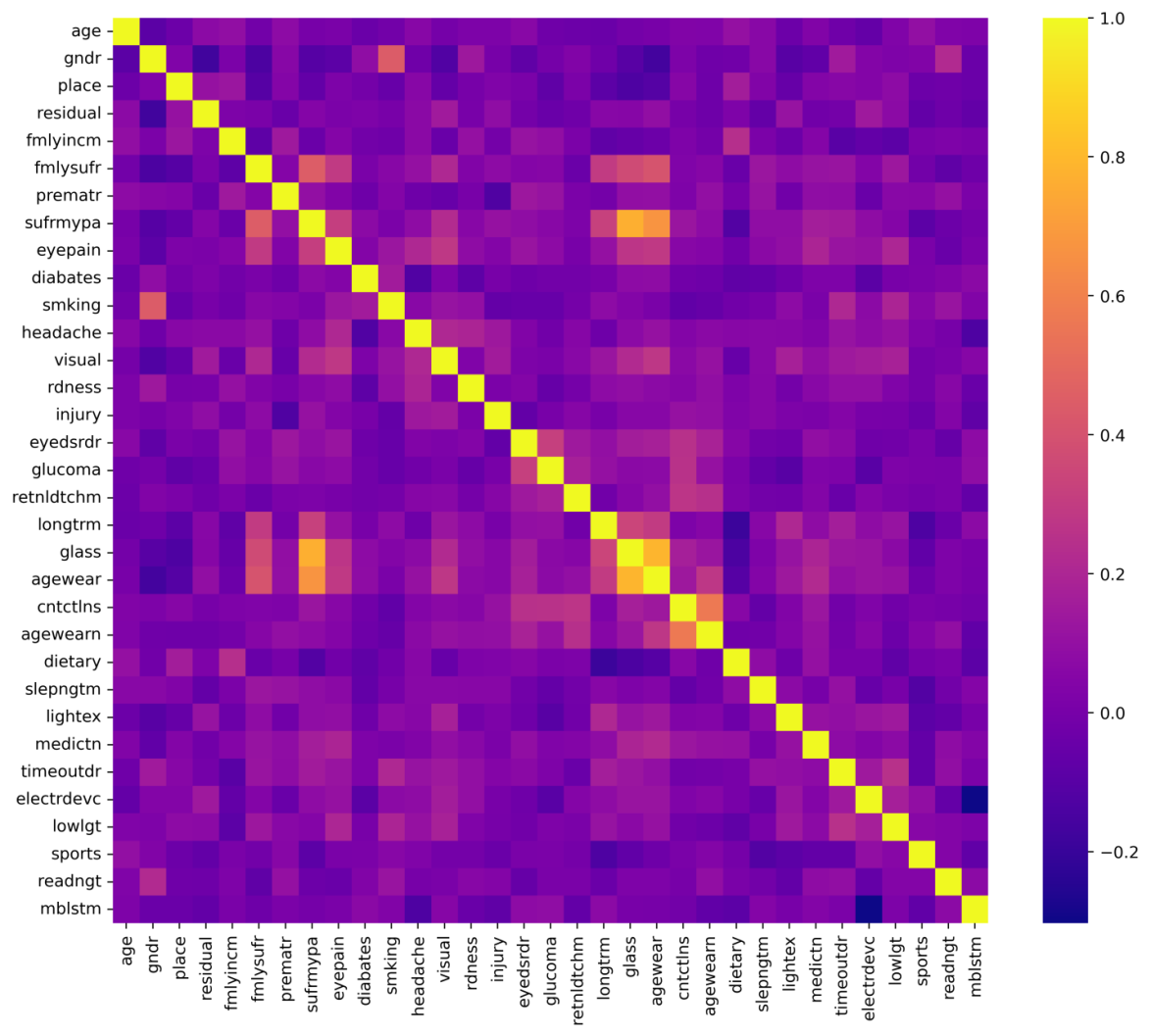  **Figure S1:** Correlation matrix plot of all variables in this study. |
| --- |

**Table S2:** Association between demographic, health variables, and lifestyle factors with myopia status among participants

| Variables | Suffering from Myopia | | | *p*-value |
| --- | --- | --- | --- | --- |
|  | Yes (n & %) | No (n & %) | Total |  |
| Age | | | | |
| <=20 years | 11 (2.1) | 11 (2.1) | 22 (4.3) | 0.732 |
| 21-24 years | 119 (23.2) | 164 (31.9) | 283 (55.1) |  |
| >=24 years | 92 (17.9) | 117 (22.8) | 209 (40.7) |  |
| Gender | | | | |
| Male | 70 (13.6) | 122 (23.7) | 192 (37.4) | **0.017** |
| Female | 152 (29.6) | 170 (33.1) | 322 (62.6) |  |
| Living place | | | | |
| Rural | 90 (17.5) | 138 (26.8) | 228 (44.4) | 0.129 |
| Urban | 132 (25.7) | 154 (30.0) | 286 (55.6) |  |
| Current residential status | | | | |
| Hall | 103 (20.0) | 121 (23.5) | 224 (43.6) | 0.612 |
| Mess | 66 (12.8) | 96 (18.7) | 162 (31.5) |  |
| Home | 47 (9.1) | 68 (13.2) | 115 (22.4) |  |
| Others | 6 (1.2) | 7 (1.4) | 12 (2.6) |  |
| Socio-economic status | | | | |
| Poor | 21 (4.1) | 26 (5.1) | 47 (9.1) | 0.105 |
| Middle | 190 (37.0) | 261 (50.8) | 451 (87.7) |  |
| Rich | 11 (2.1) | 5 (1.0) | 16 (3.1) |  |
| Any family member suffering from myopia | | | | |
| Yes | 179 (34.8) | 104 (20.2) | 283 (55.1) | **0.000** |
| No | 43 (8.4) | 188 (36.6) | 231 (44.9) |  |
| Born prematurely | | | | |
| Yes | 23 (4.5) | 15 (2.9) | 38 (7.4) | **0.025** |
| No | 199 (38.7) | 277 (53.9) | 476 (92.6) |  |
| Suffering from eyepain | | | | |
| Yes | 125 (24.3) | 75 (14.6) | 200 (38.9) | **0.000** |
| No | 97 (18.9) | 217 (42.2) | 313 (60.0) |  |
| Addicted to smoking | | | | |
| Yes | 26 (5.1) | 31 (6.0) | 57 (11.1) | 0.695 |
| No | 196 (38.1) | 261 (50.8) | 457 (88.9) |  |
| Suffering from headache | | | | |
| Yes | 118 (23.0) | 113 (22.0) | 231 (44.9) | **0.000** |
| No | 47 (9.1) | 106 (20.6) | 153 (29.8) |  |
| Sometimes | 57 (11.1) | 73 (14.2) | 130 (25.3) |  |
| Suffering from visual stress | | | | |
| Yes | 127 (24.7) | 57 (11.1) | 184 (35.8) | **0.000** |
| No | 25 (4.9) | 144 (28.0) | 169 (32.9) |  |
| Maybe | 70 (13.6) | 91 (17.7) | 161 (31.3) |  |
| Redness to eye | | | | |
| Yes | 46 (8.9) | 28 (5.4) | 74 (14.4) | **0.000** |
| No | 141 (27.4) | 234 (45.5) | 375 (73.0) |  |
| Sometimes | 35 (6.8) | 30 (5.8) | 65 (12.6) |  |
| Eye injury | | | | |
| Yes | 14 (2.7) | 8 (1.6) | 22 (4.3) | 0.053 |
| No | 199 (38.7) | 263 (51.2) | 462 (89.9) |  |
| Maybe | 9 (1.8) | 21 (4.1) | 30 (5.8) |  |
| Eye disorder | | | | |
| Yes | 28 (5.4) | 22 (4.3) | 50 (9.7) | 0.054 |
| No | 194 (37.7) | 270 (52.5) | 464 (90.3) |  |
| Glaucoma | | | | |
| Yes | 7 (1.4) | 4 (0.8) | 11 (2.1) | 0.166 |
| No | 215 (41.8) | 288 (56.0) | 503 (97.9) |  |
| Retinal detachment | | | | |
| Yes | 5 (1.0) | 5 (1.0) | 10 (2.0) | 0.661 |
| No | 217 (42.2) | 287 (55.8) | 504 (98.1) |  |
| Dietary intake | | | | |
| Poor | 28 (5.4) | 61 (11.9) | 89 (17.3) | **0.027** |
| Middle | 182 (35.4) | 222 (43.2) | 404 (78.6) |  |
| Rich | 12 (2.3) | 9 (1.8) | 21 (4.1) |  |
| Sleeping time | | | | |
| 4-6 hours | 27 (5.3) | 30 (5.8) | 57 (11.1) | 0.078 |
| 6-8 hours | 168 (32.7) | 205 (39.9) | 373 (72.6) |  |
| Above 8 hours | 27 (5.3) | 57 (11.1) | 84 (16.3) |  |
| Screen light exposure | | | | |
| Yes | 138 (26.8) | 144 (28.0) | 282 (54.9) | **0.005** |
| No | 51 (9.9) | 105 (20.4) | 156 (30.4) |  |
| Maybe | 33 (6.4) | 43 (8.4) | 76 (14.8) |  |
| Certain steroid medication | | | | |
| Yes | 38 (7.4) | 20 (3.9) | 58 (11.3) | **0.000** |
| No | 184 (35.8) | 272 (52.9) | 456 (88.7) |  |
| Insufficient time on outdoor activities | | | | |
| Yes | 122 (23.7) | 116 (22.6) | 238 (46.3) | **0.001** |
| No | 100 (19.5) | 176 (34.2) | 276 (53.7) |  |
| Spending too much time on electric devices | | | | |
| Yes | 139 (27.0) | 153 (29.8) | 292 (56.8) | 0.053 |
| No | 37 (7.2) | 69 (13.4) | 106 (20.6) |  |
| Maybe | 46 (8.9) | 70 (13.6) | 116 (22.6) |  |
| Low light work | | | | |
| Yes | 88 (17.1) | 102 (19.8) | 190 (37.0) | 0.273 |
| No | 134 (26.1) | 190 (37.0) | 324 (63.0) |  |
| Hours of reading or writing or on homework everyday | | | | |
| <1 hours | 36 (7.0) | 67 (13.0) | 103 (20.0) | 0.066 |
| 1-2 hours | 53 (10.3) | 81 (15.8) | 134 (26.1) |  |
| 3-4 hours | 79 (15.4) | 77 (15.0) | 156 (30.4) |  |
| >4 hours | 54 (10.5) | 67 (13.0) | 121 (23.5) |  |
| Daily sports activity | | | | |
| <0.5 hours | 58 (11.3) | 61 (11.9) | 119 (23.2) | 0.034 |
| 0.5-1 hours | 22 (4.3) | 54 (10.5) | 76 (14.8) |  |
| 1-2 hours | 17 (3.3) | 32 (6.2) | 49 (9.5) |  |
| >3 hours | 4 (0.8) | 6 (1.2) | 10 (1.9) |  |
| No activity | 121 (23.5) | 139 (27.0) | 260 (50.6) |  |
| Average time spending in electronic devices | | | | |
| <1 hours | 10 (1.9) | 16 (3.1) | 26 (5.1) | 0.983 |
| 1-2 hours | 25 (4.9) | 33 (6.4) | 58 (11.3) |  |
| 2-3 hours | 90 (17.5) | 116 (22.6) | 206 (40.1) |  |
| 3-4 hours | 92 (17.9) | 119 (23.2) | 211 (41.1) |  |
| >4 hours | 5 (1.0) | 8 (1.6) | 13 (2.5) |  |
